# Association of the Combined Effects between Insulin-Like Growth Factor-1 Gene Polymorphisms and Negative Life Events with Major Depressive Disorder among Chinese population in the Context of Oxidative Stress

**DOI:** 10.1155/2022/3253687

**Published:** 2022-04-22

**Authors:** Zhengxue Qiao, Yunjia Xie, Yongmei Wu, Xiuxian Yang, Xiaohui Qiu, Jiawei Zhou, Yuxin Lu, Lu Chen, Yuying Tong, Jia Xu, Jiarui Li, Jingyun He, Hui Pan, Yanjie Yang, Jiarun Yang, Tianyi Bu

**Affiliations:** ^1^Department of Medical Psychology, Harbin Medical University, Harbin, Heilongjiang Province, China; ^2^Beijing Hospital, Beijing, China; ^3^Department of Psychology, School of Education of Heilongjiang University, Harbin, Heilongjiang Province, China; ^4^The First Psychiatric Hospital of Harbin, Heilongjiang Province, China; ^5^Department of Medical Education Management, Harbin Medical University, Harbin, China; ^6^Department of Endocrinology, Peking Union Medical College Hospital, Beijing, China

## Abstract

**Background:**

Oxidative stress may be increased in a number of psychiatric disorders, including major depressive disorder (MDD). MDD has been shown to be related to insulin-like growth factor-1 (IGF-1) as well as to negative life events; exploring the interaction of IGF-1 polymorphisms and negative life events on the risk of MDD is needed. The aim of this study was to analyze the single and combined effects of IGF-1 polymorphisms (rs972936 and rs978458) and negative life events with MDD among Chinese population.

**Methods:**

420 MDD patients (according to DSM-V) and 420 age- and gender-matched control subjects were recruited in a case-control study. Negative life events were assessed using standard rating scales. IGF-1 rs972936 and rs978458 were identified by sequencing. The chi-square (*χ*^2^) tests were performed to explore the association of negative life events and IGF-1 polymorphisms with MDD.

**Results:**

Our results found that the negative life events were associated with the risk of MDD (*P* < 0.001; OR = 3.28, 95% CI: 2.19-4.85). The genotypes of IGF-1 were associated with the risk of MDD (*P* < 0.001); carrying the IGF-1 rs972936 C allele (OR = 1.53, 95% CI: 1.26-1.85) and rs978458 T allele (OR = 1.92, 95% CI: 1.58-2.34) had a higher risk of MDD. The combined effects between IGF-1 rs978458 and negative life events were associated with the risk of MDD (*P* < 0.05; OR = 2.94, 95% CI: 1.23-7.03), but IGF-1 rs972936 was not associated (*P* > 0.05).

**Conclusions:**

Based on the oxidative stress hypothesis, we confirm that carrying IGF-1 rs972936 C allele and rs978458 T allele have a higher risk of MDD and the combined effects between IGF-1 rs978458 and negative life events were associated with the risk of MDD among Chinese population.

## 1. Introduction

The major depressive disorder (MMD) is one of the most common mood disorders characterized by sadness, depression, and irritability, which affect the physical and cognitive functions of patients [1–5]. Although the etiology of MDD is not fully clear, there is increasing evidence suggesting the involvement of a specific component of metabolic stress and oxidative stress, in the pathophysiology of depression [6]. Oxidative stress refers to the biologically damaging effects of free radicals or reactive oxygen [7], which in excess cause damage to lipids, proteins, and DNA and can ultimately result in cell death. A meta-analysis pooling data from studies with different oxidative stress markers suggests that oxidative stress is increased and antioxidant defense are decreased in MDD patients [8, 9].

IGF-1 is a cytokine mainly produced by growth hormone of the liver, which is the most abundant binding protein in human blood [10]. Recent studies believe that IGF-1, also known as a brain protective factor, plays a particularly important role in the development of MDD because it protects the dopamine nervous system from oxidative stress and has antiinflammatory and antioxidant effects [11–13]. Many studies have investigated the relationship between IGF-1 levels and MDD, but there has been no consistent conclusion [14, 15]. However, prior work has shown high heritability for blood IGF-I levels (about 40–60%) [16], which is important to identify IGF-1 genetic variants [17]. We hypothesized that IGF-1 polymorphism is a risk factor for depression in the context of oxidative stress. But to date, there have been few researches on the association between IGF-1 polymorphism and MDD [11–15]. Kopczak's research revealed that 10 gene polymorphisms of the IGF-I system were nominally associated with depression susceptibility and treatment response and the statistical results were not significant in European adult whites [18]. In short, it is necessary to explore the dynamic relationship between IGF-1 polymorphism and MDD.

Researchers generally believe that both environmental and genetic factors are involved in the etiology of MDD [19]. Initially, evidence from family, twin, and adoption studies indicates that there is a significant genetic contribution to MDD [16, 20]. However, majority of individuals with a positive family history of MDD do not develop MDD [21]. Thus, the hypotheses about the role of genes for the development of MDD collectively favor the stress–diathesis theory. Some related clinical studies were subsequently carried out to support environmental factors play the key role in MDD [22–24]. Negative life events, as one of the most mature environmental risk factors for MDD, refer to loss, divorce, serious illness, interpersonal or family problems, interpersonal relations, and social difficulties [24, 25]. The latter postulates that repeated or chronic exposure of a vulnerable genotype to stressful life events may trigger the development of depression [26]. However, as far as we know, the association of the combined effects of IGF-1 polymorphisms and negative life events with MDD has not been investigated thus far. Whether it is due to IGF-1 polymorphism and its combined effects with negative life events affecting the onset of MDD in the context of oxidative stress has not been confirmed.

The aim of the present study was to test the hypothesis that the association of the combined effects between IGF-1 polymorphisms and negative life events with MDD. Toward this aim, we conducted a case-control study in Chinese population to evaluate association of the single and combined effects of IGF-1 polymorphisms and negative life events with MDD among Chinese population.

## 2. Materials and Methods

### 2.1. Participants

The study was carried out in the tertiary hospital in Harbin between 2017 and 2021, and ethical approval for all study procedures was obtained from the Harbin Medical University Ethics Committee. Trained researchers administered questionnaires to participants and all subjects gave written consent.

420 MDD participants (136 males and 284 females) were recruited through the Department of Psychiatry, a tertiary hospital in Harbin, and 420 healthy controls (158 males and 262 females) were recruited through the physical examination center of a tertiary hospital in Harbin. There were several inclusion criteria: All patients with MDD had their first onset; the ability to understand questionnaire items; Chinese version of the 24-item Hamilton Rating Scale of Depression (HRSD-24) score greater than 24 points; and underwent a structured interview by two experienced psychiatrists using the Diagnostic and Statistical Manual of Mental Disorders-fifth edition (DSM-V) to confirm MDD. Patients with clinically significant hearing or vision loss and other comorbid axis-I disorders, a family history of genetic disease, neurologic diseases, and those who received an antidepressant medication within 4 weeks were excluded from the study. Controls were matched for age, gender, and level of education with the MDD patients. The structured clinical interview for DSM-V was used to exclude psychiatric diseases, neurologic illnesses, and alcohol or drug abuse. The specific characteristics of the two groups are shown in Table 1.

### 2.2. Measures

#### 2.2.1. Negative life events

We assessed participants' negative life events with a validated Chinese version of the Life Event Scale (LES) [27], 48 items, which has been used extensively to measure life event from three domains: family life (28 items), work and study (13 items), and social and other aspects (7 items). This scale evaluates life events in this four aspects: occurrence time (unhappen = 0; a year ago = 1; one year = 2; and chronic = 3), nature (good = 1 and bad = 2), degree of mental influence (no = 0; mild = 1; moderate = 2; serious = 3; and extreme = 4), and duration of impact (three months = 1; with half a year = 2; one year = 3; and more than one year = 4). Higher scores represent higher the mental stress from negative life events. The scale has good reliability and validity in domestic and foreign researches and is widely used in genetic association studies [28, 29].

#### 2.2.2. DNA Extraction and Genotyping

Five (5) cc of venous blood was collected from all participants. Genomic DNA was extracted using the AxyPrep Blood Genomic DNA Miniprep Kit (Axygen, Union City, CA, USA). IGF-1 (rs972936 and rs978458) genotyping was performed using polymerase chain reaction (PCR). The primers used for PCR amplification were designed using Primer 5.0 software, and the specificity of a potential primer was checked using the Basic Local Alignment Search Tool (BLAST) provided by the National Center for Biotechnology Information. The primers for amplifying the IGF-1 rs972936 fragments were F 5′ TCTGGGTTAGTCATCTGTGGC 3′ and R 5′ AAGCCTAGTAGTGTGGTATGTGT 3′. The primers specific for amplification of the IGF-1 rs978458 fragments were F 5′ AAGCGAGGGTCATGCGATCTA 3′ and R 5′ TCACTCTGTTAAAGAAGCAGCCA 3′. The analysis of SNPs was performed using SNaPshot according to the manufacturer's instructions.

### 2.3. Statistical Analysis

Data were statistically analyzed using IBM-SPSS version 20.0 software. A chi-square (*χ*^2^) goodness-of-fit test was performed to test the Hardy-Weinberg equilibrium (HWE) for the genotypic distribution of SNP. The association test was done using the *χ*^2^ test. The odds ratios (OR) and their 95% confidence interval (95% CI) were calculated to identify the risk of MDD. The level of significance was employed for comparison at *P* value < 0.05.

## 3. Results

### 3.1. Association of Negative life Events and MDD

There was no significant difference between MDD patients and control subjects with respect to gender, age, marital status, and level of education (*P* > 0.05; Table 1). And people who have experienced negative life events (OR = 3.28, 95% CI: 2.19-4.85) were associated with MDD risk (*P* < 0.001; Table 2).

### 3.2. Association of IGF-1 Gene Polymorphisms on the Risk of MDD

The genotypic distributions of all selected IGF-1 polymorphisms conformed to the HWE (*P* > 0.05). The genotypes T/T, C/T, and C/C of IGF-1 rs972936 were significantly different in the case-control group (*χ*^2^ = 26.39, *P* < 0.001). There were also significant differences in the distribution of alleles T and C between the two groups difference (*χ*^2^ = 18.53, *P* < 0.001). Compared with the IGF-1 rs972936 T allele, the risk of MDD in people carrying IGF-1 rs972936 C allele increased by 1.53 times (OR = 1.53, 95% CI: 1.26-1.85). The genotypes T/T, C/T, and C/C of IGF-1 rs978458 were significantly different in the case-control distribution (*χ*^2^ = 137.51, *P* < 0.001), and the distribution of alleles T and C between the two groups was significantly different (*χ*^2^ = 42.30, *P* < 0.001). Compared with people carrying IGF-1 rs978458 C allele, the risk of MDD was increased by 1.92 times (OR = 1.92, 95% CI: 1.58-2.34) with IGF-1 rs978458 T allele. After correction by Bonferroni's multiple tests, the association between rs972936 and rs978458 of IGF-1 and MDD remained stable (*P* < 0.001) (Table 3).

### 3.3. Association of the IGF-1 rs972936 and negative life events with MDD

According to the dominant genetic model, the primitive type and mutant type of IGF-1 rs972936 were T and C, respectively, and were divided into two groups: rs972936C^−^ (TT) and rs972936C^+^ (CC + CT). The results indicated that individuals carrying the C allele of IGF-1 rs972936 which have experienced negative life events were not associated with MDD relative to the rest of the study population (*P* > 0.05) in Table 4.

### 3.4. Association of the IGF-1 rs978458 and negative life events with MDD

Also based on the dominant genetic model, the primitive type and mutant type of IGF-1 rs978458 were C and T, respectively, and were divided into two groups: rs978458T- (CC) and rs978458T+ (TT+ TC). The results showed that individuals carrying the T allele of IGF-1 rs978458 which have experienced negative life events were associated with MDD (*P* < 0.05;OR = 2.94, 95% CI:1.23-7.03) in Table 5.

## 4. Discussion

In the present study, we were able to replicate previous findings of negative life events on the risk for MDD in these participants [24]. We confirmed that IGF-1 polymorphisms (rs972936 and rs978458) are risk factors for MDD in the context of oxidative stress. We also confirmed for the single and combined effects among IGF-1 polymorphism and negative life events on the risk of MDD.

In the single loci analyses, our study found that the genotype and allele frequency of IGF-1 rs972936 and rs978458 were associated with MDD in Chinese population. People carrying the IGF-1 rs972936 C allele have a higher risk of MDD, which is 1.526 times that of those who carry the T allele, while compared with people carrying IGF-1 rs978458 C allele, the risk of MDD was increased by 1.920 times with IGF-1 rs978458 T allele. According to our review, it is the first time that the association analysis between the two IGF-1 gene loci involved in this study and MDD has been explored. In population-based studies, the research on IGF-1 gene polymorphism has focused on longevity [30], cancers [31–33], and common chronic diseases [34]. There are few studies on the association between IGF-1 gene polymorphism and MDD. A study of European white people found that the IGF-1R rs4966044 on chromosome 25 is the highest nominal associated with MDD [18]. Among neurological diseases, Alzheimer's disease also has been studied [35, 36]. IGF-1 gene polymorphism may be related to the pathophysiological mechanism of Alzheimer's, because it can regulate the cholinergic system in the brain and reduce the toxic effect of beta amyloid fragments. A study of the Han population showed that the genotype and allele frequency of IGF-1 rs972936 were significantly different between Alzheimer's disease cases and healthy controls (Pgenotype *P* = 0.006, Pallele *P* = 0.047), and compared with C alleles, people carrying the T allele of rs972936 are 1.16 times more likely to develop Alzheimer's disease [36]. This study is similar to our results, indicating that IGF-1 rs972936 is a key factor in inducing mental and cognitive diseases, but the risk allele is found to be the opposite of our research. This may be due to the fact that IGF-1 rs972936 is in MDD and Alzheimer's disease plays different roles in the development mechanism. In summary, our results found that people with IGF-1 rs972936 and rs978458 variants are more susceptible to MDD. This may be due to the mutation of IGF-1, which originally had a protective effect on MDD, failing to resist negative biological effects caused by oxidative stress.

This study further explored the association of the combined effect of IGF-1 SNPs and negative life events with MDD and found that the combined effect of individual IGF-1 rs978458 loci mutations and recent negative life events was significant associated with MDD, while IGF-1 rs979236 was not associated. However, thus far, no information on gene-environment interaction of IGF-1 in the risk of developing MDD has been found. A recent clinical study on allogeneic hematopoietic stem cell transplantation found that patients with IGF-1 rs978458 had lower maximum C-reactive protein levels after surgery, which shows that IGF-1 rs978458 is related to lower inflammation [37]. Inflammation is closely related to oxidative stress and is a well-known risk factor for MDD. It can be speculated that IGF-1rs978458 plays a more complex and important role in the etiology of MDD. Wang et al. [38] showed that patients carrying minor allele of rs972936 showed more dizziness and multiple neuropsychiatric symptoms including depression after mild traumatic brain injury. This is inconsistent with our results, which may be due to the different research objects. The present result regarding IGF-1 rs978458 is an important discovery which remains to be further verified in different regions, races, and populations. To our knowledge, this is the first study to investigate the association of the combined effects between allergic variants in IGF-1 and negative life events on susceptibility to MDD.

The results of our study should be interpreted in the context of the study's limitations. First, our study involved a limited sample of a single location in Harbin province, China. Second, the number of IGF-1 SNPs we selected is small, which might not be representative of genetic information of the gene. Third, LES was filled in subjectively by the self-reporting scale and collected retrospectively, in which related response distortions may affect the interpretability of our results. Fourth, this study was only carried out under the theoretical background of oxidative stress, without collecting the corresponding physiological indicators for in-depth exploration. Further studies are necessary to expand the gene loci to replicate our results in larger populations and other ethnic groups, combined with inflammatory cytokines and physiological indicators of oxidative stress to elucidate the underlying mechanism of gene-environment interactions involved in MDD.

## 5. Conclusions

As far as we know, this is the first report that association of the combined effects between IGF-1 gene polymorphisms and negative life events with MDD susceptibility among Chinese population in the context of oxidative stress. We found negative life events and IGF-1 polymorphisms as susceptibility factors to the risk of MDD, which are important determinants for the success in identifying genetic associations of diseases with complex characteristics. Although their specific mechanism remains to be further studied, the present results confirm that carrying IGF-1 rs972936 C allele and rs978458 T allele have a higher risk of MDD and the combined effects between IGF-1 rs978458 and negative life events were associated with the risk of MDD among Chinese population. This study provides evidence for the combined effect of IGF-1 polymorphism and environment on the mechanism of MDD and provides a basis for the clinical treatment and prevention of MDD in the future.

## Figures and Tables

**Table 1:** General demographics of the case group and the control group.

		Case (*n* = 420)	Control (*n* = 420)	*χ* ^2^	*P*
Sex	Male	136 (32.4%)	158 (37.6%)	2.53	0.112
	Female	284 (67.6%)	262 (62.4%)		
Age	16-30	90 (21.4%)	74 (17.6%)	4.90	0.179
	31-40	68 (16.2%)	84 (20.0%)		
	41-50	109 (26.0%)	116 (27.6%)		
	>50	153 (36.4%)	146 (34.8%)		
Marital status	Unmarried	60 (14.3%)	40 (9.5%)	4.58	0.101
	Married	332 (79.0%)	349 (83.1%)		
	Divorced or widowed	28 (6.7%)	31 (7.4%)		
Education	Elementary and below	89 (21.2%)	80 (19.0%)	3.47	0.483
	Junior school	96 (22.9%)	89 (21.2%)		
	Senior school	135 (32.1%)	139 (33.1%)		
	Junior college	45 (10.7%)	61 (14.5%)		
	College and above	55 (13.1%)	51 (12.2%)		

**Table 2:** Association of negative life events and MDD.

Negative life events	Case (*n* = 420)	Control (*n* = 420)	OR (95% CI)	*P*
No	320 (76.2%)	379 (90.2%)	3.28 (2.19-4.85)	**<0.001**
Yes	100 (23.8%)	41 (9.8%)		

**Table 3:** Association of IGF-1 genotypes and alleles on the risk of MDD.

SNP	Group	Genotype frequencies			*χ* ^2^ (*P* value)	Allele frequencies		*χ* ^2^ (*P* value)	OR (95% CI)
		C/C	T/C	T/T		C	T		
rs972936	Case	144 (34.3%)	205 (48.8%)	71 (16.9%)	26.39 (<0.001)	493 (58.7%)	347 (41.3%)	18.53 (<0.001)	1.53 (1.26-1.85)
	Control	120 (28.6%)	165 (39.3%)	135 (32.1%)		405 (48.2%)	435 (51.8%)		
rs978458	Case	69 (16.4%)	294 (70.0%)	57 (13.6%)	137.51 (<0.001)	432 (51.4%)	408 (48.6%)	42.30 (<0.001)	1.92 (1.58-2.34)
	Control	215 (51.2%)	133 (31.7%)	72 (17.1%)		563 (67.0%)	277 (33.0%)		

**Table 4:** Association of the IGF-1 rs972936 and negative life events with MDD.

SNP	Negative life events	Case	Control	OR (95% CI)	*P*
rs972936C^−^	No	44	122	1	
rs972936C^−^	Yes	27	13	5.76 (2.73-12.14)	<0.001
rs972936C^+^	No	232	252	2.55 (1.73-3.76)	<0.001
rs972936C^+^	Yes	117	33	0.70 (0.28-1.58)	0.358

**Table 5:** Association of the IGF-1 rs978458 and negative life events with MDD.

SNP	Negative life events	Case	Control	OR (95% CI)	*P*
rs978458T^−^	No	54	188	1	
rs978458T^−^	Yes	15	27	1.93 (0.96-3.90)	0.065
rs978458T^+^	No	222	186	4.16 (2.90-5.96)	<0.001
rs978458T^+^	Yes	129	19	2.94 (1.23-7.03)	0.015

## Data Availability

The datasets analyzed in this article are not publicly available. Requests to access the datasets should be directed to yanjie1965@163.com.
